# Supplementary material for: Effectiveness of Xpert MTB/RIF and the Line Probe Assay tests for the rapid detection of drug-resistant tuberculosis in the Central African Republic
Source: PLOS Glob Public Health. 2023 May 1;3(5):e0001847. doi: 10.1371/journal.pgph.0001847 (PMC10150968; doi:10.1371/journal.pgph.0001847)
Supplement: S1 Text — (PDF) [file pgph.0001847.s002.pdf]

PROGRAMME NATIONAL DE LUTTE CONTRE LA TUBERCULOSE

REPUBLIQUE CENTRAFRICAINE
Unité-Dignité-Travail

Bulletin de demande de microscopie / test moléculaire / culture / antibiogramme

Diagnostic et suivi de la tuberculose sensible, tuberculose pharmacorésistante (TB-PR)

Date : ___/___/_____ CDT / FOSA ____________________________________

Nom et prénom ____________________________________ Age (ans)_____ Sexe (M/F) ____

Adresse (Quartier/Village) ____________________________________

Téléphone/Email ____________________________________

Site de la TB Pulmonaire ☐ Extra pulmonaire ☐ (si extra pulmonaire, *préciser site*:________________)

Raison de l'examen :

- Diagnostic ☐
- Suivi TB sensible ☐ préciser le mois de suivi : M 2/3 ☐, ou M 5 ☐, ou M 6/8 ☐, Autres ___;
- Suivi de patient TB-RR ☐, mois M ________ N° TB-RR |__|__|__|__|__| - |__|__|__| - |__|__|__|__|-|__|__|__|

Type de patient

rechute ☐ échec de 1er traitement ☐ échec de retraitement ☐reprise ☐ contact d'un patient TB-PR ☐

autre ☐ préciser____________________

Type d'échantillon expectoration ☐ urine ☐ autre ☐ *préciser*______________

Examen demandé : Microscopie ☐ ; Xpert MTB/RIF ☐ ; Culture ☐ ; Test de sensibilité ☐ ; Test hybridation (LPA) ☐ TBLAM ☐

Demandeur: Nom: ____________________ Fonction: ______________ Signature : ________________

Résultats des tests réalisés en périphérie

Microscopie N°1 : date __________ Résultat* ________ Xpert MTB/RIF Date__________

N°2 : date __________ Résultat* ________ Résultat** MTB ___RIF ___

** N (négatif), **1-9** (rares BAAR), +, ++, +++ ** P (positif), N (négatif), I (indéterminé).*

Date__________

TB LAM	POS	NEG

Technicien de Labo: Nom: ________________ Fonction: ____________ Signature : ______________

Résultats du laboratoire de référence

Date de réception de l'échantillon_________ Numéro de culture _________

Microscopie

Négative	1-9	+	++	+++

Xpert MTB/RIF

MTB	RR

TB LAM

POS	NEG

[5] MTB P/N/I/E : P (Positif), N (Négatif), I (Indéterminé, E (Erreur/invalide)
[6] RR : O (Oui), N (Non), I (Indéterminé), E (Erreur)

Culture

Contaminée	Mycobactérie non tuberculeuse	Négative	Mycobacterium tuberculosis complex			
			1-9 colonies	10 – 100 col +	>100 - 200 col ++	> 200 col +++

Tests de sensibilité : DST et LPA

Date de collecte (compléter par le demandeur)	Méthode [a]	Numéro de série de laboratoire	Résultats [b] (pour chaque médicament)										
			R	H	E	S	Amk	Kn	Cm	___	___	___	___

[a] Préciser : DST en milieu solide ; DST en milieu liquide ; LPA 1ère ligne ; LPA 2ème ligne.

[b] Codes pour les résultats : R = résistant S = sensible C = contaminé — = test non réalisé.

Date : __________ Nom : ____________________ Fonction : ________________ Signature : ____________
