# Supplementary material for: Effectiveness of Xpert MTB/RIF and the Line Probe Assay tests for the rapid detection of drug-resistant tuberculosis in the Central African Republic
Source: PLOS Glob Public Health. 2023 May 1;3(5):e0001847. doi: 10.1371/journal.pgph.0001847 (PMC10150968; doi:10.1371/journal.pgph.0001847)
Supplement: Attachment — Submitted filename: Response to reviewers.docx [file pgph.0001847.s003.docx]

**RESPONSE TO REVIEWERS**

| **Questions des reviewer** | **Responses** |
| --- | --- |
| **Reviewer 1** | |
| 1. If a sample had an invalid Xpert result, how was this handled by the lab workflow, was it repeated, or culture done? Please clarify in the methods and any limitations | These cases have been rarely observed in our laboratory. On the rare occasions when this has occurred, the tests have been repeated on the same specimen, sometimes on a different specimen, before returning the result exactly as in the case of indeterminate results.  Culture is not performed in these cases as it is only done if rifampicin resistance has been detected by Xpert MTB/RIF.  Line 106 « invalid or indeterminated after two .. » |
| 2. A limitation in understanding the effectiveness of Xpert MTB/RIF is that culture/LPA is not done in those that are RIF negative to confirm the finding or determine if it is a false negative, and should be noted in the limitations. | Most of the algorithms proposed by WHO and adopted in NRL-TB do not allow for culture and LPA when there is no rifampicin resistance on Xpert MTB/RIF. What does allow for culture and LPA testing is really the detection of rifampicin resistance by Xpert MTB/RIF. For more clarity, we have added the following sentence:  Line 120 - 123 « According to the current NRL-TB algorithm, when the Xpert MTB/RIF detects rifampicin resistance, then the LPA tests were performed as well as the culture and susceptibility tests. If rifampicin resistance is not detected, or if the results are indeterminate or invalid, the LPA, culture and DST are not performed and the results are returned to the patient ». |
| 3. Similarly, INH resistance is only assessed in those with positive RIF, and so this will be an ongoing limitation in first-line implementation of Xpert MTB/RIF to assess for INH mono-resistance, and the authors could add this to the discussion on the implementation of MDR/XDR molecular TB testing. | The most important drug in the first-line regimen remains rifampicin, and resistance to rifampicin is sufficient to initiate second-line therapy. The interest of this issue is the decision to start second-line treatment or not and until now, INH monoresistance does not allow to change the treatment regime. Also with the introduction of GeneXpert XDR which detects INH and second line drugs, the determining factor remains the detection of rifampicin resistance with Xpert MTB/RIF. |
| 4. On Line 193-194, the authors noted “all absences” in WT8 were associated with mutation 3, but 3 had no mutation noted (Table 3). Please clarify | There were 57 cases of WT8 absence. 54 were related to MUT3 mutations and the other 3 WT8 absences had no visible mutations. There is indeed a modification of the gene, hence the appearance of WT8, but no corresponding mutation was observed, this is the explanation of the text. In these cases, sequencing would have made it possible to determine these undefined mutations. |
| 5. Why is there an inhA table but no katg table? Why not have a combined table with 2 sections? | **Why no table with katG**  We have inserted the table and you will judge its relevance « Line 222 – 224 ».  **Combined table with the 2 sections:**  On the katG gene, there is only one WT while on the inhA, there are 2 WT.  On katG, there are 2 mutations, namely MUT1 and MUT2, whereas on inhA, there are 4 mutations: MUT1, MUT2, MUT3A and MUT3B. It was therefore difficult to link them in a single table. |
| 6. In the discussion, would highlight majority of RIF resistant cases were also INH resistant, highlighting importance to consider empiric MDR treatment if Rif resistant | Your observation is correct and it has already been reported that the majority of rifampicin-resistant strains are also resistant to INH, which is why when the Xpert MTB/RIF test is positive for rifampicin resistance, the patient is considered an MDR-TB until proven otherwise. |
| 7. Was there access to the rpoB probes and mutations that were detected by Xpert to compare to LPA? | No, unfortunately, that would have been very interesting. We are thinking of including this aspect in a future study. |
| 8. Pour confirmer que tous les cas Xpert MTB/RIF positifs avec +RIF étaient également positifs à la culture sans aucune contamination ? Si oui, veuillez l'indiquer dans le texte. Ou bien les cas RIF positifs avec cultures positives ont-ils été sélectionnés pour cette analyse ? Veuillez préciser | We added the following sentence  Line 155 - 157 :  « They were performed in the level 2+ laboratory of the NRL-TB operating with a negative pressure system with dedicated Type II Microbiological Safety Stations for culture and sensitivity testing ».  Line 161 – 162 :  « Although the rate of staining has always been less than 3% in our laboratory, the rare cases when they occur are systematically repeated in order to reduce the waiting time for results ». |
| 9. As a minor comment, the focus is on RIF and INH resistance, and so the presentation of the 2nd line testing is unclear. As none were negative, would consider to just have in the results that all cases had second line testing and were MDR and none pre-xdr or xdr, and remove Figure 3 | Totally agree with you. Figure 3 is deleted.  In order to clearly state the results without any ambiguity we have detailed the results as they appear in the manuscript. Following your remark we have added the following sentence to clarify.  Line 243  « The second line LPA did not show any case of pre-XDR or XDR » |
| **Reviewer 2** | |
| 1. In the introduction the authors should justify why it is important to asses the performance of Xpert Rif detection specifically in the CAR since Xpert already has WHO approval. | In the introduction we mentioned two problems:  The first one is the long delay of the culture and the DST on solid medium which are only done at the LNT-RB in the Pasteur Institute of Bangui.  The second was the use of the Xpert MTB/RIF only at the LNR-TB, whereas this test should be deployed without delay to intensify the surveillance of MDR-TB in the country.  This study provides the NTP with scientific data on molecular tests and the importance of their contribution to the fight against tuberculosis in CAR in order to make the application of the WHO recommendations in our country effective and efficient. |
| 2. Similarly I'd like the authors to add in the discussion section what will or should change because of these results. The performance of Xpert is well documented, and unfortunately we have not been able to meaningfully lower the price of the test. So are these results useful to lobby the NRL to spend more on these tests? To confirm that they should continue to be used in the CAR? What's the next step | We have highlighted this in the conclusion which we have partially modified.  What will or must change is first of all the use of molecular tests as a first-line test for all cases of tuberculosis.  Secondly, the immediate dissemination of the tests throughout the country, which will allow the NTP to better control drug-resistant tuberculosis through a national survey of resistance to anti-tuberculosis drugs.  Line 300 – 304 in the conclusion  « The use of Xpert MTB/RIF as a first-line test has allowed the identification of MDR-TB in new patients who may have mistakenly benefited from first-line treatment and not only progressed to treatment failure but also spread MDR-TB in the community. In addition to saving considerable time, these tests are an important tool for both the diagnosis of tuberculosis and for the surveillance of MDR-TB on a national scale ». |
